# Struggles, strengths, and strategies: an ethnographic study exploring the experiences of adolescents living with an ostomy

**DOI:** 10.1186/1477-7525-6-114

**Published:** 2008-12-17

**Authors:** David B Nicholas, Sylvia R Swan, Ted J Gerstle, Theresa Allan, Anne Marie Griffiths

**Affiliations:** 1 Faculty of Social Work, Central and Northern Region, University of Calgary, #444, 11044-82 Avenue, Edmonton, Alberta, Canada; 2 Department of Social Work, Hospital for Sick Children, 555 University Ave, Toronto, ON M5G 1X8, Canada; 3 Department of General Surgery, Hospital for Sick Children, 555 University Ave, Toronto, ON M5G 1X8, Canada; 4 5B Ward – Long Stay Surgical Unit, Hospital for Sick Children, 555 University Ave, Toronto, ON M5G 1X8, Canada; 5 Gastroenterology, Hepatology and Nutrition, Hospital for Sick Children, 555 University Ave, Toronto, ON M5G 1X8, Canada

## Abstract

**Background:**

Adolescents with IBD requiring ostomy surgery experience perioperative needs that may exceed those of patients experiencing other major abdominal surgery [1]. This procedure requires ongoing and vigilant daily care and management. Gastrointestinal symptoms and complications impose psychological and social stresses on young patients [2], and the procedure results in body image changes and daily regimens of self-care. This study aimed to explore adolescents' experiences and quality of life following ostomy surgery.

**Methods:**

Ethnographic interviews and a subsequent focus group were conducted with 20 adolescents with an ostomy or j-pouch being treated at the Hospital for Sick Children in Toronto, Canada. Interviews were transcribed verbatim and subjected to theme generation.

**Results:**

Findings suggest that adolescents are profoundly affected by their ostomy. Adolescents convey strength as well as adjustment struggles. Identified impacts include body intrusion and body image changes, decreased independence, secrecy about the ostomy, adjustment over time, challenges for the family, and strategies for constructively moving forward.

**Conclusion:**

Implications address the importance of ensuring meaningful opportunities to understand and reframe the stresses of illness. An ongoing clinical challenge involves the promotion of a healthy self-esteem and psychosocial adjustment for these adolescents and their families. Finding effective ways to minimize stress and embarrassment and reframe personal shame, constitute important clinical priorities. Opportunities for peer support and family dialogue may assist in clarifying worries and easing the burden carried by these young persons. Flexible and adequately funded resources are advocated in fostering quality of life.

## Background

The incidence of inflammatory bowel disease (IBD) varies globally, and ranges from 0.3% to 0.8% of the population in northern Europe, Scandinavia, New Zealand and the United States [3]. IBD is reported to manifest during childhood or adolescence in 20–25% of patients [2,4]. Canadian data estimates that 12% of individuals in the 17–24 year age group with ulcerative colitis require surgery [5], and approximately 26% of children with moderate to severe ulcerative colitis require surgery within 5 years of diagnosis because of treatment failure or an inability to control symptoms [6,7]. An ostomy, also commonly called a stoma, is a surgically created opening through which a portion of the small or large intestine is exteriorized for the diversion of fecal matter outside the body [8]. In some cases, the ostomy eventually will be closed and a surgically-created reservoir called a j-pouch will be created from the small intestine. Adolescents with IBD who require ostomy surgery experience perioperative needs that may exceed that of patients experiencing other major abdominal surgery [1]. This procedure requires ongoing and vigilant daily care and management. Gastrointestinal symptoms and complications impose psychological and social stresses on young patients [2], and the procedure results in body image changes and daily regimens of self-care.

This interpretive study examined adolescents' experiences following ostomy surgery. An ethnographic approach, using interviews and a focus group, was used to identify impacts of the ostomy including challenges, resources and adaptational strategies.

Relatively little is known about the psychosocial adjustment of young patients with IBD [9]. Clinical experience and exploratory research suggest that this population is at emotional risk due to body image issues, periodic illness exacerbation, complications, daily care requirements, uncertainties, developmental concerns, and stigma [10]. IBD patients who undergo ostomy surgery experience further psychosocial obstacles as surgery exceeds the demands and challenges typically faced by individuals with IBD[10]. Several studies with small sample sizes suggest that these adolescents experience substantial post-surgical challenges with respect to diminished self esteem, peer socialization, social stigma, sexual identity, independence, body image shifts, embarrassment, grief, and loss of control [1,11-15]. Post-surgical adolescent ostomy patients are further reported to identify self-repulsion when initially viewing their stoma [11].

Ostomy surgery often occurs during adolescence, a time in human development when peer acceptance and body image issues may be particularly heightened [1,11,12,15]. Several studies particularly identify body image concerns among these adolescents such as insecurities about height, weight and personal appearance [2,14,15]. Strategies for improving the psychosocial wellbeing of adolescents with an ostomy have been offered in the literature. Erwin-Toth [15] interviewed adults who had undergone ostomy surgery between the ages of 6 and 12 years. Informants retrospectively described negative impacts on their lives during adolescence and recommended peer contact with other adolescents experiencing an ostomy as a means for managing stress. Adolescents are reported to counteract the challenges of living with an ostomy through self-management, social support, a positive attitude, and increased control over their own care [16].

Peer support is reported to be a positive moderator of coping among adolescents. Moreover, supportive and accessible resources are a priority; however, exploratory research is yet needed to examine key issues and needs of these young ostomy patients. While limited exploratory evidence exists, current studies are limited in volume and methodological rigor. Clinical evidence is largely drawn from anecdotal accounts and often comprises mixed samples of children and adolescents. To address this gap and specifically explore the experiences of youth, this qualitative study examined post-ostomy experiences of adolescents. Specifically, the study sought the illumination of perceptions, challenges, resources, and adaptational strategies of adolescents who had undergone ostomy surgery.

## Methods

This exploratory ethnographic study, as part of a larger intervention-based project, incorporated qualitative interviews and a follow-up focus group. Ethnography is a well-established qualitative approach that effectively explores the lived experience of a population [17,18]. Ethnography invites the identification of daily processes, routines, events and perspectives from the vantage point of participants. It is characterized by prolonged engagement and open discussion within a population, yielding rich description reminiscent of the population and area under study. Interviews and focus groups are commonly used within this research approach. Accordingly, the face-to-face interviews used in this study offered in-depth narrative detail, and the focus group data added triangulation, corroboration, elaboration and clarification [19]. This focus group offered notable data yield because peer interaction appeared to be highly valued by participants. They openly shared and disclosed common experiences. Interviews and the focus group were semi-structured, using an interview schedule in which open-ended questions guided the interviewing process. The interview guide reflected existing literature and team members' clinical experience in pediatric ostomy nursing, social work, medicine and surgery. Interviews lasted approximately one hour, and the focus group was 1.5 hours. They were audio recorded and transcribed verbatim. Written consent was obtained from all participants. Ethics approval was obtained from the Research Ethics Board at the Hospital for Sick Children prior to study commencement.

### Data Analysis

Transcripts were subjected to content analysis, which comprised (i) line-by-line review of codes within transcripts, (ii) analysis of similarity and difference within transcript codes, (iii) organization of codes in the development of categorization schemes, and (iv) solidification of themes through review of codes relative to transcripts [20]. Computer software for qualitative data analysis was utilized to assist with code identification, category development, and theme generation. Trustworthiness of emerging themes was ensured through established means of demonstrating referential rigor, including adequacy, negative case analysis, and peer debriefing [21]. Educative authenticity, an emerging criterion of qualitative research rigor, refers to direct educational benefits to participants following study involvement [22]. This outcome was achieved through the contribution of data in the ultimate development of a participant-based teaching booklet for newly diagnosed adolescents facing ostomy surgery. Verbatim text quotes, including those presented herein, may also appear in this educational resource targeted to adolescent ostomy patients.

### The Sample

Participants were recruited from a database of adolescents with an ostomy who had participated in a support intervention offered at a large pediatric hospital in central Canada. A total of twenty adolescents were interviewed, and all were subsequently invited to a follow-up focus group, of which seven were able to attend. Eleven females and nine males participated in the study, and participants were culturally diverse, geographically scattered from both urban and rural home locations, and they ranged in terms of represented family constellation. The mean age of participants was 15.3 years, and participants ranged from 13 to 19 years of age. Mean time since surgery was 2.9 years with a range of three months to ten years; and participants had an ostomy or j-pouch.

## Results and Discussion

Participants conveyed common experiences and impacts related to their IBD and ostomy. They repeatedly stated that their daily lives had been dramatically altered, and the ostomy had introduced struggles as well as elements promoting personal growth and maturity. Identified themes comprise body intrusion and body image changes, decreased independence, secrecy about the ostomy, adjustment over time, challenges for the family, and strategies in constructively 'moving forward'. Each of these identified themes is explored below.

### Body Intrusion and Body Image Challenges

Adolescents identified self-consciousness regarding body image changes and the intrusion upon personal body space. Difficulties adjusting to body changes were described. A participant reminisced about pre- and post-surgery adjustments associated with stoma by stating: "I just wanted to ...close my eyes, then open them and... 'hey it's done'... It was this massive swollen thing. I was grossed out by it. But I eventually got used to looking at it." While body image was of concern to participants, they increasingly became accustomed to, and less repelled by, the appearance of the stoma.

Adolescents described intrusion of their 'personal space' and bodies due to frequent health examinations and medical procedures. An adolescent consequently described frustration and remarked, "...so many people in the hospital touched me when I didn't want to be touched. Like the doctors poked me on my stomach". The participants identified self-consciousness and worry that others would notice their ostomy; a concern that they described as embarrassing and, in some cases, humiliating. One adolescent stated, "At first I was so self-conscious of it... If I needed to empty it or something, I thought, 'oh my God, people are staring at me'." Adolescents worried that peers would become aware of the ostomy, as exemplified below.

"When we dance now,... I have a problem with that. It's so wrong, it's so weird. I'm just so afraid because it's kind of 'feelable', like the plastic ring. So I'm just afraid that someone is going to feel it and say 'what is that?'."

-----

"...one of my friends...went like this to my stomach and one of (her) fingers was right on it. I cried when I went home from school for so long because in my head I was like 'oh my God, (she) felt it..."

### Decreased Independence and Control

Upon becoming ill, participants were subjected to increased levels of personal care and involvement by parents. Participants' independence, a coveted element in adolescence, decreased as they physically needed a parent's help. In some cases, parents' heightened concern for the adolescent's well-being incited increased parental scrutiny toward, and intimate care of, the youth, as illustrated below.

"My parent was constantly on me. My advice to a teen about having the operation is to expect your parent or parents to basically be overprotective more than they usually are, because they will be. And for me, ...my mom grew out of it...I think my mom was just really, really worried about me to begin with, and whether I'd be able to deal with it. If I'd be okay. If I'd still be as sick, because I was really sick before I had the ostomy. After my mom saw that I could deal with it, it basically became 'okay, the teen can deal with it. I don't need to help the teen anymore'."

-----

"Right away my mom was so protective of me and she was always worried about stuff. Whether I was drinking enough, because you can get so dehydrated... I think it's just the way parents are. They're our protectors and they see themselves as that."

While adolescents appreciated their parents' support, some stated that parents often had difficulty relinquishing care to the adolescent. Parental concerns and stresses were recognized as significant which, in some cases, caused what was viewed to be over-involvement in the adolescent's activities of daily living: "After I got (ill), it was ... frustrating because ...my family and extended family...treated me like a china doll ...like 'don't eat that, don't do that, careful'."

### Secrecy: Considerations in Deciding Whether to Tell Others About the Ostomy

Participants described a personal struggle over disclosing the ostomy to others. They appeared to experience shame and embarrassment about the ostomy and, in varying degrees, hesitated to convey this information. Vulnerability and embarrassment were heightened by the personal nature of the ostomy and its association with toileting.

Participants described a process of coming to a sense of "okayness" with the ostomy. This process appeared necessary in ultimately being able to unashamedly divulge the presence of the ostomy to others. For some, this confidence came with relative ease yet, for most, the process was substantially difficult. Fear of peer stigma or non-acceptance was a consuming concern. A participant spoke about his process of becoming comfortable disclosing the ostomy to others:

"I think that before you tell anybody, you have to be comfortable with it yourself. And people see that. If you're comfortable with it, they don't freak out as much. If you're just like, 'oh yeah, I have this' and you brush it off like it's nothing, then they won't freak out as much. But if it's like some big mystery, like 'oh my god, what do they have?' Then they start thinking weird things."

Adolescents described careful consideration of *who *they would tell about the ostomy. An impediment against revealing this information was described as embarrassment or shame. Conversely, the decision to share this information was often made when the adolescent expected that the receiver would respond favorably and supportively: "I think you've really got to choose the people that you know will be supportive, the people that you know are your friends." Deciding to inform peers presented a harrowing conundrum as participants agonized over how and when to tell others about the ostomy. After informing others, participants generally seemed to be relieved. Acceptance and support by a peer were celebrated, which, in turn, increased the adolescents' own acceptance of their ostomy:

"There is one person that I freaked out about telling, but I did. And (he) understood completely and I think (he) was one of the people who took it the best under the circumstances. So it is surprising what you can really find out about people when you tell them something like that."

-----

"And just follow your heart with telling people. Just take a chance... I think it was really important to me to tell people and let everybody know that you do have bad days."

Beyond ambivalence then relief surrounding disclosure, participants used this process of truth-telling to ultimately determine the extent and value of their friendship with the recipient of the information. They appeared to have come to a new realization that others' acceptance or rejection of their information was not a reflection of the ill adolescent's 'problem', but rather an indication of the other person's virtue as a friend. Accordingly, friendships were cemented or distanced based on respective positive or negative responses.

"It's not so bad because I wouldn't want to hang around somebody who thinks 'oh (she) has something gross' or 'let's not hang around with (her)'. So you find other friends who can take you for who you are, on the inside rather than the outside. That's better than the other people who can't accept it."

Participants appeared to have shifted from an externalized locus of control in which they feared negative reprisals to increased internalized control in which they validated themselves as persons of worth. And in some cases, they had come to celebrate this growth in themselves as a result of their adjustment. One participant affirmed his worthiness, stating:

"There (are) lots of chances in this life. So you take a chance on somebody and if they don't want to talk to you anymore then it's their loss because they are missing out on a really good person."

### Adjustment Over Time

Adolescents developed mastery, confidence, and independence as they managed their ostomy care over time. Initially, the ups and downs of daily care were upsetting, but participants described gradually developing methods of ostomy management.

"At first everything you do will cause a problem. After a while you get used to it, you understand it. You understand how it works with you. Every person is different, so you can't ask this person and say, 'I changed it every certain amount of days,' because some people are going to have more leaks and some ...aren't going to be able to cope for a week. But it's a lot of figuring out how it works for you."

Helpful resources and strategies fostering adaptation and mastery of self-care were garnered, as illustrated by participant comments.

"...you have to educate yourself and know the products out there because what's right for you might not be right for someone else... I just stick with what the hospital gave me...But it's just a matter of trying it and seeing what is best for you..."

-----

"...the first leak is always going to be the worst one ever. Not necessarily in amount, but it is going to be so awful. 'It's leaking. What am I going to do?' It's awful. And you're going to freak out, but it's not so bad. Leaks will happen, there is no way you can really avoid them. Sometimes you go for years without leaks, sometimes every other day you'll have a leak. It's not so bad. Clean up, change, done."

Emotional and psychological movement toward acceptance and accommodation were not identified to follow a predictable time course across all participants. Accordingly, this process of adjustment occurred more gradually and was a more difficult process for some than for others. Several teens appeared more at ease in talking about their journey as well as the presence of the ostomy. These data did not focus on differences between an ostomy and j-pouch and the role of ostomy closure in this process of adjustment. Accordingly, while several participants expressed relief when the ostomy had been closed, their discussion tended to reflect processes and experiences with the ostomy.

### Challenges for the Family

The illness and ostomy were described to have a vicarious impact on participants' families. Participants identified specific difficulties faced by their families, as illustrated by one adolescent: "I felt that (my family) was unhappy that this was happening to me, that something was wrong with me...it's just this look, when they first found out I was going to have the operation, it was just this look they had every time they came in to visit me". Realizing the physical demands and stresses faced by parents, one adolescent said, "I think my advice to parents is that it's okay to feel helpless sometimes. It's okay to feel that you're not superman or superwoman, and that you can't do everything all the time."

Participants also conveyed sadness and concern about the impact of their condition and care needs on their healthy siblings.

"My mother almost spent every time in the hospital with me. She stayed over with me at the hospital... And while it is nice to have someone with you at all times, she was there on (a special day) with me, yet the other kids were at home without my mother. It was hard on them."

-----

"I think for siblings, it is really difficult. You really need to talk to them and make them feel as if they are needed, and to address their problems. When you have an illness in the family it takes away from a lot of the other siblings, and they also need care."

Participants sometimes appeared to blame themselves for their parents' lack of time for healthy siblings. As an example, an adolescent reflected on a hospitalization, and placed responsibility upon himself for the deprivation of the healthy sibling's time with the parent.

"One time I went to the hospital, I was really sick. I had a high fever, and I knew I was going to be awhile.... My brother came, and said 'bye mom, see you in a month.' And that was really hard for me...because it felt like I was taking my mom away for a month. I need my mom, but I tell my brother every single time we talk, 'if I'm sick or whatever, I'm sorry, I don't mean to do this'."

### Sources of Strength: Family and Friends

Despite worrying about the impact on family and friends, participants recognized their own need for ongoing support from family. Physical help was required by participants during times of illness, as was ongoing emotional support, as exemplified below.

"I find that I get a lot of strength from my family...After my surgery I remember (some close family members) looked at my stoma and they were like 'oh, it's kind of cute'."

-----

"When I'm in the hospital, my brother is always on the phone with me saying 'how are you, are you okay?' Every night it's on the phone for hours talking, telling me what I missed on TV that day or whatever."

-----

"I get a lot of help, strength from ... my friends."

In some cases, the depth of participants' friendships intensified, particularly as friends provided support, surveillance and/or care for the adolescent with the ostomy and j-pouch. As an example, a participant described the role of her friends during times of recreation.

"I even go swimming with my friends and they basically keep an eye on me. To begin with, I empty it out before I start swimming... But when I start moving around everybody knows that I start digesting food. So they just keep an eye on it for me and if it gets really bad, they tell me and I just do something about it. So it kind of helps having someone look at it for you."

### Benefits and Growth

Realization that many people generally care and are willing to support the adolescent, were insights that fostered emotional strength and encouragement. Several participants stated that the ostomy allowed them to discover or re-align important life perspectives and priorities. They felt that they had become more sensitive and accepting of themselves and others. Participants felt that this heightened awareness may have been accelerated by their illness and ostomy experiences. One adolescent concluded,

"Overall, I'm just really happy that this happened to me. The first person that I actually told that to said 'okay, you're insane'. But honestly, thinking about (it), I'm so happy it happened to me. It really made me sit back and look at my life and put things into perspective and it actually helped me make a career decision because I know what I want to go into...now. And I just find I'm an overall happier person even though it happened to me because it's just nice knowing that I have this and people still see me as a normal person. And it's nice to know that people out there are good and nice. I don't find it that bad of a thing."

-----

'The ostomy makes you a better person. It makes you more understanding in a way because you want to be treated with respect, so you treat people with respect as well."

-----

"I feel like I kind of go with the flow more now, you know. Whatever comes up I can handle it..."

Adolescents identified adaptive strategies and means of coping with their circumstances. Some identified the importance of being honest about personal feelings, emotions and needs, as illustrated below.

"...sometimes it's healthy to cry, I don't know if it was the way that I was brought up, but somehow I got the mentality that I'm constantly supposed to be happy and I'm not allowed to have a bad day. So when I actually figured out, I thought, 'yeah I had a bad day, yeah I need to cry today and just let it all out.' And I think having an ostomy, sometimes you just have to do that, because sometimes you'll have a bad day where it leaks on you. You put a second one on, that one leaks and then you go to a third one, and you're like 'dear God, please don't let this one leak'. So after that happens, just take some time and relax."

-----

"I think that it is normal for anybody to have trouble accepting it (the ostomy). But I think ... it gets to a certain point where you probably have to move on a certain bit, even look at the small steps towards getting to the good part of it. You're hoping something good is going to come of it: I'm going to feel better, I'm going to be able to do lots of things. So accepting it, you know, you grieve for a little while if you may, if that's what you want to call it."

Social support was seen to be crucial in moving forward and feeling less isolated. Peer support emerged as a key resource in coping.

"I definitely think getting some of your fears out or anything that you want to talk about... Talking to your friends can really help. It's also good to talk to your parents or any other person that you feel will listen. But I feel that talking to your friends is really good, especially people that are in your school, which is basically two thirds of your life... Basically...they are probably the ones that really know you best for two thirds of the year."

-----

"I think with the new teens that are going in today, they should talk to somebody who already went through it. But sometimes, people just aren't going to try to talk to somebody. I know some kids who don't want to talk to anyone because they are too shy. But I found, on the Internet, it is easier to open up to people. You can ask the embarrassing questions that you didn't want to ask. They can't see you. They don't know who you are. But you can ask them basically anything."

Adjusting to life with an ostomy ultimately allowed adolescents to develop skills which were perceived to promote personal growth. Educating themselves about the ostomy by talking with health care professionals and/or reading books, pamphlets, or Internet resources helped participants become informed and involved in their own care. Using these various resources and supports, participants described a growing conviction that the ostomy was manageable and, as such, they sought to, "live life to the fullest". Accordingly, they offered positive perspectives and recommendations for adaptation and growth.

"Live life to the fullest. You only have one life. It's short. It can be bittersweet. Do what you can to make life good for yourself."

-----

"Basically...think positive... Even if the challenges (associated with the ostomy) seem like they will never end, they will end at some point. So just keep looking for that point and it will come."

-----

"... life is whatever you make out of it. You look at the glass either half full or half empty. It's just your outlook on life. You can either go into your operation, 'oh my God, this is the worst thing ever'. Or this is basically like the best it can get, it can't get any worse from here. You've reached the bottom of whatever you reach and the only way left is up."

As exemplified here, these participants had accumulated skills and insights over time that allowed them to gain personal satisfaction and growth in their lives. Despite struggles, participants generally viewed the experience of the ostomy to have added value to their life and increased their compassion for others. They described this to have followed sufficient, although varying, time for reflection and assimilation of the illness in daily life, and they identified family and peer support as a means of facilitating this growth.

## Conclusion

A range of struggles, strengths and strategies were identified by participants. They experienced psychological pain, which, in some cases, resulted from self-consciousness and shame. They dealt with substantial health struggles yet simultaneously identified personal growth. Realizing the potential for growth that comes with adversity may offer hope for patients, families, and health care providers.

Toward this end, supports were clearly helpful. Parents, siblings and friends appeared to be vital resources in contributing to participants' well-being. Yet concern for family members was also identified. Participants worried about the vicarious impact of the illness on their family which was sometimes expressed in terms of personal reprisal. Negative impact on the family, including siblings' receipt of less parental attention, was sometimes assumed by the teen with the ostomy; however, such concern appeared to be worked through and framed as uncontrollable consequences of their condition.

Notwithstanding this apparent resolution, ensuring meaningful opportunities to redress challenging perceptions of illness and its impact, merit consideration in assessment and intervention. An ongoing clinical challenge involves the promotion of healthy self-esteem and psychosocial adjustment. Finding effective ways to minimize stress and embarrassment and reframe personal shame, clearly constitute potential areas of clinical pursuit and program development. Opportunities for peer support and family dialogue may assist in clarifying worries and easing the burden carried by these young patients. Flexibility of resources in allowing a family-centered approach and the development of constructive responses appear merited.

Beyond clinical implications in fostering adaptation, further research needs to address the processes of adjustment to an ostomy and clinical means to support adaptation among teens and their family. Also, increased public education about pediatric IBD and living with an ostomy, may be of benefit in advancing community and societal knowledge and understanding. Finding ways to advance public awareness, promote adaptation, and diminish personal stigma constitute important clinical, health policy and research priorities. This invites multifaceted approaches including direct resources to patients and families, further research, and widespread knowledge translation approaches at both practice and policy levels. Given the profound impact of an ostomy on adolescents and their families, finding ameliorative strategies and resources clearly emerge as aims worthy of pursuit.

## Competing interests

The authors declare that they have no competing interests.

## Authors' contributions

DN, Senior Associate Scientist, Research Institute, The Hosptal for Sick Children, led this research initiative. As researcher and former social worker in gastroenterology, DN brought both psychosocial research experience and a strong clinical understanding of social support and adjustment issues often experienced by adolescents with an ostomy. With his clinical and research background, DN contributed project leadership, facilitation, data analysis, and manuscript preparation.

SS is an experienced social worker in the Division of General Surgery. To the study, SS brought expertise in psychosocial interventions for adolescents with an ostomy and their families. SS brought to this study clinical understanding about the needs of this adolescent population as well as an extensive awareness of support issues and resources. Also, SS was integral in all phases of the study.

TG, staff surgeon in the Division of General Surgery, SickKids, attends to surgical needs of adolescent ostomy patients. TG particularly contributed to this study as an expert advisor regarding the clinical and surgical needs of patients.

TA, a Registered Nurse in Enterostomal Therapy, provides pre- and post-surgical care to adolescents with an ostomy. To the study, TA brought extensive expertise and experience in the information and support needs of this population, and was involved in various elements of the study.

AMG is a staff gastroenterologist in the Division of Clinical Nutrition and Gastroenterology. AMG brought a demonstrated clinical expertise and research background in quality of life of children and adolescents with Inflammatory Bowel Disease.
